# Graduate studies in Collective Health in Brazil: trajectories, evaluation, and challenges

**DOI:** 10.1590/0102-311XEN023226

**Published:** 2026-03-23

**Authors:** Bernardo Lessa Horta, Aylene Bousquat, Alberto Novaes Ramos, Carmem E. Leitão Araujo, Rita Barata Barradas, Guilherme Loureiro Werneck, Romulo Paes-Sousa

**Affiliations:** 1 Programa de Pós-graduação em Epidemiologia, Universidade Federal de Pelotas, Pelotas, Brasil.; 2 Faculdade de Saúde Pública, Universidade de São Paulo, São Paulo, Brasil.; 3 Faculdade de Medicina, Universidade Federal do Ceará, Fortaleza, Brasil.; 4 Faculdade de Ciências Médicas, Santa Casa de São Paulo, São Paulo, Brasil.; 5 Instituto de Estudos em Saúde Coletiva, Universidade Federal do Rio de Janeiro, Rio de Janeiro, Brasil.; 6 Instituto René Rachou, Fundação Oswaldo Cruz, Belo Horizonte, Brasil.

Each civilizational leap achieved in Brazilian history, from extending social rights to disease control and expanding strategic technologies, was preceded by consistent investments in education, science, technology, and innovation. In the field of Health, this translates into the ability to produce knowledge, train people, guide policies, plan and manage systems, master critical technologies, and respond to emergencies autonomously ^1^ ^,^ ^2^ ^,^ ^3^. Graduate studies in Collective Health, in its academic and professional formats, is part of a democratic infrastructure that sustains the Brazilian Unified National Health System (SUS, acronym in Portuguese): it is the space in which research meets the public health system and in which innovation is transformed into concrete improvement of comprehensive care for people. Responsibly valuing and evaluating these graduate studies in Collective Health is, therefore, to dispute and invest in the present and future of the country, in the quality of care, and in health sovereignty ^1^ ^,^ ^2^ ^,^ ^3^.

Collective Health as a constituted field and creation of the Brazilian Association of Collective Health (Abrasco, acronym in Portuguese) in 1979 occurred in lockstep with the Health Reform movement and democratic struggles, as they opposed authoritarian and mercantilist health projects in favor of a health project as a right and a universal public system ^2^ ^,^ ^4^ ^,^ ^5^ ^,^ ^6^. From the first national graduate meetings, even before Abrasco, the area has structured a critical reflection on education, training, curricula, profile of graduates and social insertion, articulating self-evaluation and external evaluation as regulation and defense instruments of its own academic-political project ^4^ ^,^ ^5^ ^,^ ^6^. Subsequent readings of the field, which discuss its axes or subareas, spaces, and “countenances”, reinforce the idea of a dynamic area, marked by disputes and reshaping, but anchored in the defense of health as a right and democracy as a founding value ^6^ ^,^ ^7^ ^,^ ^8^ ^,^ ^9^.

Graduate studies in Collective Health makes up the scope of national graduate studies, sharing several challenges and achievements. The 2025-2029 Brazilian National Graduate Plan (PNPG, acronym in Portuguese) updates diagnoses and recommendations for the Brazilian National Graduate System (SNPG, acronym in Portuguese), emphasizing social impact, integration with production systems, strengthening professional and network formats, and reducing regional asymmetries ^10^. PNPG recognizes that the expansion of Brazilian graduate studies is marked by territorial inequalities, periods of austerity and defunding, productivist pressures, and recurrent threats to social rights and democracy. These contexts have been evinced in recent historical phenomena such as the COVID-19 pandemic, misinformation, and attacks on science and public universities ^1^ ^,^ ^3^ ^,^ ^10^. Graduate studies in Collective Health anticipates this agenda by: (1) addressing socially relevant problems, (2) articulating training and research with SUS services and policies, and (3) fostering cooperation networks that disseminate competencies across the national territory ^3^ ^,^ ^5^ ^,^ ^11^ ^,^ ^12^. Studies on graduates, program performance, and relationship with SUS suggest a positive correlation between graduate studies in Collective Health vitality and the ability to formulate, implement, and evaluate public health policies in different federative contexts ^11^ ^,^ ^12^ ^,^ ^13^. These studies have evinced the relevant insertion of graduates in teaching, research, management, and health services, confirming graduate studies in Collective Health’s strategic role in consolidating the SUS and in building a more equitable and democratic society ^11^ ^,^ ^12^.

In 2025, the area achieved the historic mark of 101 Graduate Programs (PPGs, acronym in Portuguese), fruit of a trajectory that began in the 1970s and sped up in recent decades ^4^ ^,^ ^5^ ^,^ ^6^ ^,^ ^11^. This quantitative growth was accompanied by qualitative maturation: the area consolidated itself as a scientific field, producing knowledge to qualify the SUS; institutionalize practices, technologies, and public policies; encompassing the complex relations of the social determination of the health-disease process in human collectivities ^5^ ^,^ ^6^ ^,^ ^7^ ^,^ ^11^ ^,^ ^12^. A very successful project, its growth and recognition go beyond national borders, inserting Brazilian production into global debates on social justice, democracy, and the right to access to health.

To answer the theoretical and methodological challenges of our health-disease-care object, the area assumes an interdisciplinary character anchored on three major discursive fields: Epidemiology; Social and Human Sciences in Health; and Health Policy, Planning, and Management. This composition renews and articulates objects, methods, techniques, and practices to understand and intervene in the health-disease processes considering their multiple dimensions ^2^ ^,^ ^5^ ^,^ ^7^ ^,^ ^8^ ^,^ ^9^. Although each of these axes presents its own epistemological, methodological and technical specificities, the coexistence between subfields has enabled Collective Health to remain cohesive around the health-disease-care object in populations and the health project as a public good ^6^ ^,^ ^7^ ^,^ ^8^ ^,^ ^9^.

Producing masters and PhDs is central to Brazil, being numerically and qualitatively expressive. Recently, many of its graduates have occupied key positions in universities, research institutes, health services, social movements, and management bodies ^5^ ^,^ ^6^ ^,^ ^7^ ^,^ ^11^ ^,^ ^12^ ^,^ ^13^. Moreover, this process valued the integration between education, training, research, and concrete realities of health management and care, producing applied knowledge geared towards qualifying public policies and strengthening SUS ^3^ ^,^ ^11^ ^,^ ^12^ ^,^ ^13^.

Academic and professional training are indispensable formats for the graduate studies in Collective Health’s mission to be fully realized. Academic programs train faculty and researchers who lead research groups, developing theories and methods, and producing evidence that structures the field and critically feeds back into health policies, with relevant weight in the world science scenario ^5^ ^,^ ^6^ ^,^ ^7^ ^,^ ^11^. Professional programs combine scientific training and strategic or technological research geared towards solving concrete problems, bringing issues closer to health services through robust methodologies, generating responses in shorter cycles and strengthening SUS’ institutional capacities ^3^ ^,^ ^11^ ^,^ ^12^. This is not a question of hierarchy, but of complementarity: the academic format further develops the scientific and critical basis of the field, while the professional format accelerates the translation, circulation, and social appropriation of knowledge. Both depend on stable institutional environments, adequate funding, and recognition of their specificities in evaluation ^10^ ^,^ ^11^.

Programs from all areas undergo a rigorous evaluation system managed by the Brazilian Coordination for the Improvement of Higher Education Personnel (CAPES, acronym in Portuguese), responsible for assessing program performance, guiding their improvement and establishing criteria for their offer. Over the last decades, the evaluation processes have been adjusted to solve the obstacles to the full development of Brazilian graduate studies, going through different phases and emphases: faculty qualification; reduction of program semesters; increase in the dissemination of scientific production. Excessive emphasis on this last dimension, however, has contributed to productivism, the precariousness of academic work and the proliferation of unoriginal studies which negatively impact graduate education and the capacity of research to question relevant problems within the SUS and society. In this sense, introducing and improving qualitative criteria in the evaluation process are central - and the graduate studies in Collective Health is one of the CAPES evaluation areas with the greatest theoretical and operational accumulation in this direction ^5^ ^,^ ^6^ ^,^ ^9^ ^,^ ^11^. As Cameron’s text, which is usually attributed to Einstein, suggests: “*not everything that can be counted counts, and not everything that counts can be easily counted*”, which does not mean that what matters is no longer evaluated; it means, rather, disputing which criteria guide the evaluation.

With these concerns as a backdrop, the Quadrennial Evaluation process (2021-2024) was conducted in September 2025, in Brasilia, with participation of about 100 consultants who analyzed the programs based on criteria and indicators established for the field. In Collective Health, the evaluation parameters are widely discussed with legitimate representations of the field itself, particularly within the scope of the Abrasco Forum of Graduate Coordination, respecting the collective construction and social control over the graduate policy ^4^ ^,^ ^6^ ^,^ ^11^.

The evaluation presents three central requirements: the program itself, the educational process, and the impacts generated. The capacity of the programs to produce changes in local, regional, national, and international realities is valued, contributing to expand the power of Brazilian graduate studies as a structuring force of the SUS and social protection policies.

In the Area Document for 2025-2028, Collective Health together with CAPES outlines and updates the strategic paths for the quadrennium ^10^ ^,^ ^11^. An important inflection is underway: less productivism and more public relevance from each program. Emphasis is put on qualified intellectual production, valuing rigorous quantitative and qualitative approaches, conveyed in good journals and books with substantive criteria (ethical publishing practices, in addition to impact) ^9^ ^,^ ^11^, without forgetting the technical-technological production capable of informing practices and policies. In this perspective, the field solidifies its commitment to Open Science, encouraging transparency, reproducibility, intra- and transdisciplinary collaboration, reducing barriers to access to knowledge, including publication in open repositories, expanding the visibility and public utility of the results ^10^ ^,^ ^11^. An internationalization agenda centered on South-South solidarity cooperation and horizontal partnerships is also underscored, refusing models subordinated to rankings and metrics that have little dialogue with the needs of SUS and society.

Analyses of graduate studies in Collective Health show that it shares merits and problems with science and technology in Brazil, cautioning about the risk of naturalizing inequalities and losing sight of the public meaning of scientific production ^5^ ^,^ ^11^ ^,^ ^12^. Recent studies on the performance of Collective Health graduate programs and SUS development reiterate this warning, showing that programs located in more vulnerable contexts tend to face greater barriers to achieving the same levels of productivity required in consolidated centers, despite playing a decisive role in training staff and responding to local needs ^11^ ^,^ ^12^ ^,^ ^13^. The evaluation, therefore, must recognize the plurality of projects, the diversity of products and the distinct insertions in different regional and institutional contexts, without renouncing strict quality criteria ^10^ ^,^ ^11^. In political terms, it is a matter of resisting the homogenization that penalizes peripheral programs and of affirming that reducing regional asymmetries is an inseparable part of the agenda on academic excellence.

Beyond counting articles and the number of citations, evaluation must capture the capacity of the programs to: innovate in the articulation between teaching, research, and outreach; to produce qualified staff committed to the SUS; dialogue with society and generate solid knowledge that supports redistributive and social protection policies ^1^ ^,^ ^2^ ^,^ ^3^ ^,^ ^11^ ^,^ ^12^. The Area Document explains the notion of “excellence with a public sense” and reaffirms the tradition of scientific excellence, combined with a commitment to health policies, advocating internationalization based on solidarity-based, non-subordinate cooperation with verifiable impacts ^1^ ^,^ ^4^ ^,^ ^10^ ^,^ ^11^. For the graduate studies in Collective Health, this means affirming the inseparability between academic quality, commitment to the SUS, and the defense of democracy, in a global context marked by health, environmental, economic, and political crises ^1^ ^,^ ^2^ ^,^ ^3^ ^,^ ^10^ ^,^ ^11^ ^,^ ^12^. In times of advancing authoritarian and denialist agendas, sustaining this tripod is an explicitly political choice.

Self-assessment and strategic planning processes are key to achieving this agenda. Previous evaluations have already pointed to self-evaluation as a little institutionalized dimension, often restricted to the bureaucratic filling out of forms, with a weak link between the situational diagnosis, academic community participation and management decisions ^11^ ^,^ ^12^ ^,^ ^13^. In the new cycle, programs are expected to plan their development in an articulated manner with the Institutional Development Plan, define impact indicators, and consolidate participatory self-assessment routines involving professors, students, technicians and institutional partners ^10^ ^,^ ^11^. By strengthening the culture of internal evaluation, the programs also reinforce democratic practices of academic management, in line with the history of Collective Health as a field committed to participation and social control in health ^1^ ^,^ ^2^ ^,^ ^4^ ^,^ ^6^.

In educational processes, network, cooperation, and solidarity initiatives such as interinstitutional master’s degree (Minter, acronym in Portuguese), interinstitutional doctorate (Dinter, acronym in Portuguese), interinstitutional consortia, and thematic networks, become central instruments to reduce asymmetries and create critical mass in strategic areas, especially in the North, Central-West, and Northeastern countryside ^3^ ^,^ ^4^ ^,^ ^10^ ^,^ ^11^ ^,^ ^12^. Inductive measures should prioritize qualified expansion, strengthening of local networks, and the opening of vacancies and lines geared towards regional needs of the SUS, with responsible involvement of programs offered by institutions with greater capacity to obtain resources for research and technological development, which act as articulating poles of cooperative networks ^3^ ^,^ ^4^ ^,^ ^10^ ^,^ ^11^ ^,^ ^12^. Recent initiatives to train and support PPG coordination in Collective Health, such as the 1st Course to Strengthen Graduate Studies in Brazilian Collective Health conducted by Abrasco, CAPES and the Brazilian Ministry of Health, with a special focus on programs with grades 3 and 4, point to concrete ways to strengthen the field by highlighting structural barriers, mapping needs and building paths to internalize excellence, recognizing their potential to strengthen the SUS in different territories.

Abrasco works to consolidate a continuous agenda of support, strengthening, and articulation of graduate studies in Collective Health in Brazil, recognizing and valuing the diversity that characterizes the field and its potential to contribute to the SUS ^4^ ^,^ ^6^ ^,^ ^11^.

Another crucial challenge concerns the social and racial composition of graduate studies. Despite advances in affirmative action policies, graduate studies in Collective Health still reflects structural inequalities in tertiary education system and in Brazilian society, with people from the popular classes - especially Black, Indigenous, *quilombolas*, traditional populations, and other racialized groups -, LGBTQIA+, people with disabilities being underrepresented among students, professors, and research leaders. Consolidating a graduate program committed to democracy and to reducing inequities presupposes incorporating explicit goals of racial and gender equity in the selection of students and professors, in the distribution of grants and in the research agenda, in dialogue with social movements and with policies to promote racial equality in Brazil ^1^ ^,^ ^2^ ^,^ ^4^ ^,^ ^6^ ^,^ ^10^. Graduate studies in Collective Health that intend to be critical and transformative cannot reproduce, within its programs, the same exclusionary structure that it seeks to confront in the territories.

Graduate studies in Public Health are not a superfluous good, but a strategic asset of Brazil, as has become increasingly evident in the response to the COVID-19 pandemic, in the defense of science, and in the support of the SUS against attack on social policies ^1^ ^,^ ^2^ ^,^ ^3^. It is a condition for a more problem-solving SUS, for an education system and for science, technology and innovation committed to the public interest and, above all, for a fairer, healthier, and more democratic society ^1^ ^,^ ^2^ ^,^ ^3^ ^,^ ^4^ ^,^ ^10^ ^,^ ^11^ ^,^ ^12^. To this end, it requires: stable funding, responsible evaluation, solidarity between programs, and national and international cooperation. Ultimately, the future of the graduate studies in Collective Health goes hand in hand with that of Brazilian democracy. To strengthen one is to defend and reinvent the other.
