# Supplementary material for: Graduate studies in Collective Health in Brazil: trajectories, evaluation, and challenges
Source: Cad Saude Publica. 2026 Mar 23;42:e00023226. doi: 10.1590/0102-311XEN023226 (PMC13011255; doi:10.1590/0102-311XEN023226)
Supplement: Translation

# Translation: Pós-graduação em Saúde Coletiva no Brasil: trajetórias, avaliação e desafios

**DOI:** 10.1590/0102-311XPT023226

**Authors:** Bernardo Lessa Horta, Aylene Bousquat, Alberto Novaes Ramos, Carmem E. Leitão Araujo, Rita Barata Barradas, Guilherme Loureiro Werneck, Romulo Paes-Sousa

**Affiliations:** 1 Programa de Pós-graduação em Epidemiologia, Universidade Federal de Pelotas, Pelotas, Brasil.; 2 Faculdade de Saúde Pública, Universidade de São Paulo, São Paulo, Brasil.; 3 Faculdade de Medicina, Universidade Federal do Ceará, Fortaleza, Brasil.; 4 Faculdade de Ciências Médicas, Santa Casa de São Paulo, São Paulo, Brasil.; 5 Instituto de Estudos em Saúde Coletiva, Universidade Federal do Rio de Janeiro, Rio de Janeiro, Brasil.; 6 Instituto René Rachou, Fundação Oswaldo Cruz, Belo Horizonte, Brasil.

Na história brasileira, cada salto civilizatório alcançado, da ampliação de direitos sociais ao controle de doenças e à expansão de tecnologias estratégicas, foi precedido por investimentos consistentes em educação e ciência, tecnologia e inovação. No campo da saúde, isso se traduz na capacidade de produzir conhecimento, formar pessoas, orientar políticas, planejar e gerir sistemas, dominar tecnologias críticas e responder a emergências com autonomia ^1^ ^,^ ^2^ ^,^ ^3^. A pós-graduação em Saúde Coletiva, em suas modalidades acadêmica e profissional, faz parte de uma infraestrutura democrática que sustenta o Sistema Único de Saúde (SUS): trata-se do espaço em que a pesquisa encontra o sistema público de saúde e em que a inovação se transforma em melhoria concreta, do cuidado integral às pessoas. Valorizar e avaliar, de forma responsável, a pós-graduação em Saúde Coletiva é, portanto, disputar e investir no presente e no futuro do país, na qualidade do cuidado e na soberania sanitária ^1^ ^,^ ^2^ ^,^ ^3^.

A constituição do campo da Saúde Coletiva e a criação da Associação Brasileira de Saúde Coletiva (Abrasco), em 1979, ocorreram em sintonia com o movimento da Reforma Sanitária e as lutas democráticas, ao se contrapor a projetos autoritários e mercantilistas de saúde em favor de um projeto de saúde como direito e de um sistema público universal ^2^ ^,^ ^4^ ^,^ ^5^ ^,^ ^6^. Desde os primeiros encontros nacionais de pós-graduação, ainda antes da criação da Abrasco, a área estruturou uma reflexão crítica sobre formação, currículos, perfil de egressos(as) e inserção social, articulando a autoavaliação e a avaliação externa como instrumentos de regulação e de defesa de um projeto acadêmico-político próprio ^4^ ^,^ ^5^ ^,^ ^6^. Leituras posteriores sobre o campo, que discutem seus eixos ou subcampos, espaços e “semblantes”, reforçam a ideia de uma área dinâmica, marcada por disputas e recomposições, mas ancorada na defesa da saúde como direito e da democracia como valor fundante ^6^ ^,^ ^7^ ^,^ ^8^ ^,^ ^9^.

A pós-graduação em Saúde Coletiva insere-se no âmbito da pós-graduação nacional, partilhando diversos desafios e conquistas. O Plano Nacional de Pós-Graduação (PNPG) 2025-2029 atualiza diagnósticos e recomendações para o Sistema Nacional de Pós-Graduação (SNPG), enfatizando o impacto social, a integração com os sistemas produtivos, o fortalecimento de modalidades profissionais e em rede e a redução de assimetrias regionais ^10^. O PNPG reconhece que a expansão da pós-graduação brasileira é marcada por desigualdades territoriais, períodos de austeridade e desfinanciamento, pressões produtivistas e ameaças recorrentes a direitos sociais e à própria democracia. Esses contextos foram evidenciados em fenômenos históricos recentes, como: a pandemia de COVID-19, a desinformação e ataques à ciência e às universidades públicas ^1^ ^,^ ^3^ ^,^ ^10^. A pós-graduação em Saúde Coletiva antecipa essa agenda ao: (1) trabalhar problemas socialmente relevantes; (2) articular a formação e a pesquisa aos serviços e às políticas do SUS; e (3) estimular redes de cooperação que disseminam competências pelo território nacional ^3^ ^,^ ^5^ ^,^ ^11^ ^,^ ^12^. Estudos sobre egressos(as), o desempenho de programas e a relação com o SUS sugerem uma correlação positiva entre a vitalidade da pós-graduação em Saúde Coletiva e a capacidade de formular, implementar e avaliar políticas públicas de saúde em diferentes contextos federativos ^11^ ^,^ ^12^ ^,^ ^13^. Esses estudos têm evidenciado a inserção relevante de egressos(as) em docência, pesquisa, gestão e serviços de saúde, confirmando o papel estratégico da pós-graduação em Saúde Coletiva na consolidação do SUS e na construção de uma sociedade mais equânime e democrática ^11^ ^,^ ^12^.

Em 2025, a área alcançou a marca histórica de 101 programas de pós-graduação (PPGs), resultado de uma trajetória iniciada nos anos 1970 e acelerada nas últimas décadas ^4^ ^,^ ^5^ ^,^ ^6^ ^,^ ^11^. Esse crescimento quantitativo veio acompanhado de amadurecimento qualitativo: a área consolidou-se como campo científico, produzindo conhecimento para qualificar o SUS; institucionalizar práticas, tecnologias e políticas públicas; compreendendo as complexas relações da determinação social do processo saúde-doença em coletividades humanas ^5^ ^,^ ^6^ ^,^ ^7^ ^,^ ^11^ ^,^ ^12^. Esse projeto tem sido muito bem-sucedido e o crescimento e reconhecimento da área ultrapassam as fronteiras nacionais, inserindo a produção brasileira em debates globais sobre justiça social, democracia e direito à saúde.

Para responder aos desafios teóricos e metodológicos do nosso objeto saúde-doença-cuidado, a área assume caráter interdisciplinar, ancorando-se em três grandes formações discursivas: Epidemiologia; Ciências Sociais e Humanas em Saúde; e Política, Planejamento e Gestão em Saúde. Essa composição renova e articula objetos, métodos, técnicas e práticas para compreender e intervir nos processos saúde-doença em suas múltiplas dimensões ^2^ ^,^ ^5^ ^,^ ^7^ ^,^ ^8^ ^,^ ^9^. Embora cada um desses eixos tenha suas especificidades epistemológicas, metodológicas e técnicas, a convivência entre subcampos tem permitido que o campo se mantenha coeso em torno do objeto saúde-doença-cuidado em populações e de um projeto de saúde como bem público ^6^ ^,^ ^7^ ^,^ ^8^ ^,^ ^9^.

A formação de mestres e doutores(as) é central para o país, sendo numericamente e qualitativamente expressiva. Nas últimas décadas, muitos de seus egressos têm ocupado posições-chave em universidades, institutos de pesquisa, serviços de saúde, movimentos sociais e órgãos de gestão ^5^ ^,^ ^6^ ^,^ ^7^ ^,^ ^11^ ^,^ ^12^ ^,^ ^13^. Ademais, esse processo valorizou a integração entre formação, pesquisa e realidades concretas da gestão e da atenção em saúde, produzindo conhecimentos aplicados orientados à qualificação de políticas públicas e ao fortalecimento do SUS ^3^ ^,^ ^11^ ^,^ ^12^ ^,^ ^13^.

As modalidades de formação acadêmica e profissional são indispensáveis para que a missão da pós-graduação em Saúde Coletiva se expresse integralmente. Os programas acadêmicos formam quadros docentes e pesquisadores(as) que lideram grupos de pesquisa, desenvolvem teorias e métodos, e produzem evidências que estruturam o campo e retroalimentam criticamente as políticas de saúde, inclusive com relevante peso no cenário da ciência mundial ^5^ ^,^ ^6^ ^,^ ^7^ ^,^ ^11^. Os programas profissionais combinam formação científica e pesquisa estratégica ou tecnológica orientada à solução de problemas concretos, aproximando questões dos serviços de saúde por meio de metodologias robustas, gerando respostas em ciclos mais curtos e fortalecendo capacidades institucionais do SUS ^3^ ^,^ ^11^ ^,^ ^12^. Não se trata de hierarquia, mas de complementaridade: a modalidade acadêmica aprofunda a base científica e crítica da área, enquanto a profissional acelera a tradução, a circulação e a apropriação social do conhecimento; ambas dependem de ambientes institucionais estáveis, de financiamento adequado e de reconhecimento de suas especificidades na avaliação ^10^ ^,^ ^11^.

Os programas de todas as áreas estão submetidos a um rigoroso sistema de avaliação, gerenciado pela Coordenação de Aperfeiçoamento de Pessoal de Nível Superior (CAPES), responsável por aferir o desempenho dos cursos, orientar seu aprimoramento e estabelecer critérios para sua oferta. Ao longo das últimas décadas, os processos avaliativos foram ajustados para solucionar os entraves para o pleno desenvolvimento da pós-graduação brasileira, passando por distintas fases e ênfases: qualificação do corpo docente; redução dos tempos de formação; e aumento da veiculação da produção científica. A ênfase excessiva nesta última dimensão, entretanto, contribuiu para o produtivismo, a precarização do trabalho acadêmico e a proliferação de estudos sem originalidade, com impacto negativo sobre a formação de pós-graduandas(os) e sobre a capacidade da pesquisa de interpelar os problemas relevantes do SUS e da sociedade. Nesse sentido, a introdução e o aperfeiçoamento de critérios qualitativos no processo avaliativo são centrais - e a pós-graduação em Saúde Coletiva é uma das áreas de avaliação da CAPES com maior acúmulo teórico e operacional nessa direção ^5^ ^,^ ^6^ ^,^ ^9^ ^,^ ^11^. Como sugere o texto de Cameron, que costuma ser atribuído à Einstein: “*nem tudo o que pode ser contado conta, e nem tudo o que conta pode ser facilmente contado*”, o que não significa que o que importa deixe de ser avaliado; significa, sim, disputar quais critérios orientam a avaliação.

Com estas preocupações como pano de fundo, procedeu-se, em setembro de 2025, em Brasília, ao processo de Avaliação Quadrienal (2021-2024), com a participação de cerca de cem consultores(as), que analisaram os programas com base em critérios e indicadores estabelecidos para área. Na Saúde Coletiva, os parâmetros de avaliação são amplamente discutidos com representações legítimas do próprio campo, particularmente no âmbito do Fórum de Coordenações de Pós-Graduação da Abrasco, respeitando a construção coletiva e o controle social sobre a política de pós-graduação ^4^ ^,^ ^6^ ^,^ ^11^.

Há três quesitos centrais na avaliação: o programa em si, o processo de formação e os impactos gerados. No conjunto, valoriza-se a capacidade dos programas de produzir mudanças nas realidades locais, regionais, nacionais e internacionais, contribuindo para ampliar a potência da pós-graduação brasileira como força estruturante do SUS e de políticas de proteção social.

No Documento de Área para 2025-2028, a Saúde Coletiva junto à CAPES demarca e atualiza os caminhos estratégicos para o quadriênio ^10^ ^,^ ^11^. Está em curso uma inflexão importante: menos produtivismo e mais relevância pública do trabalho de cada programa. A ênfase recai na produção intelectual qualificada, valorizando abordagens quantitativas e qualitativas rigorosas, veiculada em bons periódicos e livros com critérios substantivos (práticas éticas de editoração, para além do impacto) ^9^ ^,^ ^11^, sem esquecer da produção técnico-tecnológica capaz de informar práticas e políticas. Nesta perspectiva, a área aprofunda o compromisso com a Ciência Aberta, com estímulo à transparência, à reprodutibilidade, à colaboração intra e transdisciplinar, à redução das barreiras de acesso ao conhecimento, incluindo a publicação em repositórios abertos, ampliando a visibilidade e a utilidade pública dos resultados ^10^ ^,^ ^11^. Também se enfatiza uma agenda de internacionalização centrada em cooperação solidária Sul-Sul e em parcerias horizontais, recusando-se a modelos subordinados a rankings e métricas que pouco dialogam com as necessidades do SUS e da sociedade.

Análises da pós-graduação em Saúde Coletiva mostram que a área partilha méritos e problemas com o conjunto da ciência e da tecnologia no país, alertando para o risco de naturalizar desigualdades e de perder de vista o sentido público da produção científica ^5^ ^,^ ^11^ ^,^ ^12^. Estudos recentes sobre o desempenho da pós-graduação em Saúde Coletiva e o desenvolvimento do SUS reiteram esse alerta, ao mostrar que programas localizados em contextos mais vulneráveis tendem a enfrentar maiores barreiras para alcançar os mesmos patamares de produtividade exigidos nos centros consolidados, embora desempenhem papel decisivo na formação de quadros e na resposta a necessidades locais ^11^ ^,^ ^12^ ^,^ ^13^. A avaliação, portanto, deve ser capaz de reconhecer a pluralidade de projetos, a diversidade de produtos e as inserções diferenciadas em contextos regionais e institucionais distintos, sem renunciar a critérios rigorosos de qualidade ^10^ ^,^ ^11^. Em termos políticos, trata-se de resistir à homogeneização que penaliza programas periféricos e de afirmar que reduzir as assimetrias regionais faz parte, de forma inseparável, da agenda de excelência acadêmica.

Mais do que contabilizar artigos e o número de citações, importa captar a capacidade dos programas de: inovar na articulação entre ensino, pesquisa e extensão; formar quadros qualificados e comprometidos com o SUS; dialogar com a sociedade; e produzir conhecimento sólido que sustente políticas redistributivas e de proteção social ^1^ ^,^ ^2^ ^,^ ^3^ ^,^ ^11^ ^,^ ^12^. O Documento de Área explicita a noção de “excelência com sentido público” e reafirma a tradição de excelência científica, combinada ao compromisso com políticas de saúde, defendendo a internacionalização baseada em cooperação solidária, não subordinada, com impactos verificáveis ^1^ ^,^ ^4^ ^,^ ^10^ ^,^ ^11^. Para a pós-graduação em Saúde Coletiva, isso significa afirmar a indissociabilidade entre a qualidade acadêmica, o compromisso com o SUS e a defesa da democracia, em um contexto global marcado por crises sanitárias, ambientais, econômicas e políticas ^1^ ^,^ ^2^ ^,^ ^3^ ^,^ ^10^ ^,^ ^11^ ^,^ ^12^. Em tempos de avanço de agendas autoritárias e negacionistas, sustentar esse tripé é uma escolha explicitamente política.

Processos de autoavaliação e de planejamento estratégico são peças-chave para concretizar essa agenda. Avaliações anteriores já apontavam a autoavaliação como uma dimensão pouco institucionalizada, muitas vezes restrita ao preenchimento burocrático de formulários, com fraca vinculação entre o diagnóstico situacional, participação da comunidade acadêmica e as decisões de gestão ^11^ ^,^ ^12^ ^,^ ^13^. No novo ciclo, ganha centralidade a expectativa de que os programas planejem seu desenvolvimento de forma articulada ao Plano de Desenvolvimento Institucional, definam indicadores de impacto e consolidem rotinas de autoavaliação participativa, envolvendo docentes, discentes, técnicos(as) e parceiros institucionais ^10^ ^,^ ^11^. Ao fortalecer a cultura de avaliação interna, os programas também reforçam práticas democráticas de gestão acadêmica, em sintonia com a história da Saúde Coletiva como campo comprometido com a participação e controle social em saúde ^1^ ^,^ ^2^ ^,^ ^4^ ^,^ ^6^.

Nos processos formativos, iniciativas em rede, cooperação e solidariedade, como Mestrado Insterinstitucional (Minter), Doutorado Insterinstitucional (Dinter), consórcios interinstitucionais e redes temáticas, tornam-se instrumentos centrais para reduzir assimetrias e criar massa crítica em áreas estratégicas, especialmente nas regiões Norte, Centro-oeste e no interior do Nordeste ^3^ ^,^ ^4^ ^,^ ^10^ ^,^ ^11^ ^,^ ^12^. Medidas indutivas devem priorizar a expansão qualificada, o adensamento de redes locais e a abertura de vagas e linhas orientadas às necessidades regionais do SUS, com envolvimento responsável de programas situados em instituições com maior capacidade de obtenção de recursos para pesquisa e desenvolvimento tecnológico, que atuem como polos articuladores de redes cooperativas ^3^ ^,^ ^4^ ^,^ ^10^ ^,^ ^11^ ^,^ ^12^. Iniciativas recentes de formação e apoio a coordenações de PPGs em Saúde Coletiva, como o 1º Curso de Fortalecimento da Pós-Graduação em Saúde Coletiva Brasileira, realizado por Abrasco, CAPES e Ministério da Saúde, com foco especial em programas com notas 3 e 4, apontam caminhos concretos para o fortalecimento da área, ao evidenciar barreiras estruturais, mapear necessidades e construir trilhas de interiorização da excelência, reconhecendo o potencial desses programas para o fortalecimento do SUS em diferentes territórios.

A Abrasco atua na consolidação de uma agenda contínua de apoio, fortalecimento e articulação da pós-graduação em Saúde Coletiva no Brasil, reconhecendo e valorizando a diversidade que caracteriza o campo e seu potencial de contribuição para o SUS ^4^ ^,^ ^6^ ^,^ ^11^.

Outro desafio crucial diz respeito à composição social e racial da pós-graduação. Apesar de avanços em políticas de ações afirmativas, a pós-graduação em Saúde Coletiva ainda reflete desigualdades estruturais do sistema de educação superior e da sociedade brasileira, com sub-representação de pessoas oriundas das classes populares, sobretudo negras, indígenas, quilombolas, populações tradicionais e outros grupos racializados, LGBTQIA+, pessoas com deficiência entre discentes, docentes e lideranças de pesquisa. A consolidação de uma pós-graduação comprometida com a democracia e com a redução de iniquidades supõe incorporar metas explícitas de equidade racial e de gênero na seleção de estudantes e docentes, na distribuição de bolsas e na agenda de pesquisa, em diálogo com movimentos sociais e com as políticas de promoção da igualdade racial no país ^1^ ^,^ ^2^ ^,^ ^4^ ^,^ ^6^ ^,^ ^10^. Uma pós-graduação em Saúde Coletiva que se pretende crítica e transformadora não pode reproduzir, no interior de seus programas, a mesma estrutura de exclusão que busca enfrentar nos territórios.

A pós-graduação em Saúde Coletiva não se trata de um bem supérfluo, mas um ativo estratégico do Brasil, como se tornou ainda mais evidente na resposta à pandemia de COVID-19, na defesa da ciência e na sustentação do SUS em momentos de ataque às políticas sociais ^1^ ^,^ ^2^ ^,^ ^3^. É condição para um SUS mais resolutivo, para um sistema de educação e de ciência, tecnologia e inovação comprometido com o interesse público e, sobretudo, para uma sociedade mais justa, saudável e democrática ^1^ ^,^ ^2^ ^,^ ^3^ ^,^ ^4^ ^,^ ^10^ ^,^ ^11^ ^,^ ^12^. Para tanto, requer: financiamento estável, avaliação responsável, solidariedade entre programas e cooperação nacional e internacional. Em última instância, o futuro da pós-graduação em Saúde Coletiva acompanha o da democracia brasileira. Fortalecer uma é, ao mesmo tempo, defender e reinventar a outra.
